# Sperm Protein 17 Expression by Murine Epithelial Ovarian Cancer Cells and Its Impact on Tumor Progression

**DOI:** 10.3390/cancers10080276

**Published:** 2018-08-20

**Authors:** Qian Gao, Sue D. Xiang, Kirsty Wilson, Mutsa Madondo, Andrew N. Stephens, Magdalena Plebanski

**Affiliations:** 1 Department of Immunology and Pathology, Central Clinical School, Faculty of Medicine, Nursing and Health Science, Monash University, Melbourne, Victoria 3004, Australia; qgao2012@sina.com (Q.G.); Kirsty.Wilson@monash.edu (K.W.); mutsamadondo@gmail.com (M.M.); 2 Department of Clinical Laboratory, Xiangya Hospital, Central South University, Changsha 410008, China; 3 Centre for Cancer Research, Hudson Institute of Medical Research, Clayton, Victoria 3168, Australia; Andrew.N.Stephens@hudson.org.au; 4 Department of Molecular and Translational Sciences, Monash University, Clayton, Victoria 3168, Australia; 5 School of Health and Biomedical Sciences, RMIT University, Bundoora, Victoria 3083, Australia

**Keywords:** sperm protein 17 (Sp17), ID8, PD-L1, STAT3, MHC II, Paclitaxel, tumor resistant, immune evasion

## Abstract

The cancer testis antigen sperm protein 17 (Sp17) is a promising antigenic target in epithelial ovarian cancer (EOC) vaccine development. However, its role in ovarian cancer is unclear. We isolated and expanded Sp17^+^ and Sp17^−^ clones from the murine EOC cell line ID8, and compared their in-vitro cell growth characteristics and in-vivo tumorigenicity. We also examined the potential co-expression of molecules that may influence cancer cell survival and interaction with immune cells. These include stimulatory and immunosuppressive molecules, such as major histocompatibility class I molecules (MHC I), MHC II, cytotoxic T lymphocyte associated antigen-4 (CTLA-4), CD73, CD39, tumor necrosis factor receptor II (TNFRII), signal transducer and activator of transcription 3 (STAT3) and programmed death-ligand 1 (PD-L1). Whilst the presence of Sp17 was not correlated with the ID8 cell proliferation/growth capacity in vitro, it was critical to enable progressive tumor formation in vivo. Flow cytometry revealed that Sp17^+^ ID8 cells displayed higher expression of both STAT3 and PD-L1, whilst MHC II expression was lower. Moreover, Sp17^high^ (PD-L1^+^MHCII^−^) cell populations showed significantly enhanced resistance to Paclitaxel-induced cell death in vitro compared to Sp17^low^ (PD-L1^−^MHCII^+^) cells, which was associated in turn with increased STAT3 expression. Together, the data support Sp17 as a factor associated with in-vivo tumor progression and chemo-resistance, validating it as a suitable target for vaccine development.

## 1. Introduction

Epithelial ovarian cancers (EOCs) are the most lethal of all gynecological malignancies, with about 70% five-year mortality [1]. Initially responsive to platinum-based chemotherapy, >90% of patients will subsequently develop recurrent, platinum-resistant disease, at which point therapeutic options are limited. Emerging immunotherapies have demonstrated durable and clinically robust responses in many solid tumor types (e.g., melanoma, non-small cell lung cancer); however, cancers like EOC are typically defined by limited treatment options and poor overall prognosis (reviewed in [2]). Despite well-established correlations between immune cell infiltrate, prognosis, and the demonstrated antigenicity of multiple tumor-associated targets (e.g., NY-ESO-1, Mesothelin, Her2/Neu, TP53, MUC16 and others [3,4,5,6]), clinical trials targeting these antigens have shown very limited efficacy [2]. Currently, there are a number of promising immunotherapies targeting checkpoint inhibitors that have been trialed for the treatment of EOC, such as anti-programed cell death protein-1 (PD-1) antibodies (e.g., Nivolumab and Pembrolizumab), as well as anti-programmed death-ligand 1 (PD-L1) antibody (e.g., Avelumab); although promising, but only showed limited efficacy (response rate between 9% and 15%), at least in the context of EOC [2]. The development of therapeutic vaccines, whereby an appropriately adjuvanted antigenic peptide (epitope), DNA, or ex-vivo pulsed dendritic cells are delivered in vivo to stimulate immune activation, represents an alternative approach for EOC management. Indeed, vaccination has been proven to be highly successful for other gynecological tumors (e.g., cervical cancer) [2]; in EOC, however, vaccine development has been hampered by the lack of identified tumor-specific antigens. Clearly, the selection of appropriate target antigens is critical for the design of effective vaccine platforms [7].

Cancer testes antigens (CTAs) have received much attention as potential targets for cancer immunotherapy. Normally restricted to embryonic/placental tissues and testes, CTAs are expressed in the vast majority of EOCs [8]. Sperm protein 17 (Sp17) is a 151 amino acid (aa) CTA with high inter-species homology [9]. In normal tissues Sp17 plays a key role in the interaction of sperm with the zona pellucida during fertilization [10], and promotes heparin sulfate-mediated cell–cell adhesion [9]. In EOCs, however, the role of Sp17 is unclear. Several studies have suggested roles in cell migration, drug resistance, and metastasis [11], and Sp17 expression is correlated with chemoresistance in clear-cell adenocarcinoma [12]. Similar to other CTAs, Sp17 displays restricted expression in healthy tissues, except in male spermatozoa, and limited expression in embryonic germinal cells and ciliated epithelia cells [13,14], but displays high expression in cancer [15]. Moreover, over 90% of vasectomized males develop natural immunity to Sp17, suggesting it as a safe antigenic target for vaccine design [16]. Combined with its cancer-specific expression, Sp17 has therefore been suggested as a promising target for ovarian cancer immunotherapy and vaccine development [14,16,17].

The expression of immune-inhibitory molecules on tumor cells plays an important role in establishing and maintaining an immunosuppressive environment within EOCs. EOC cells express key elements of the immune checkpoint pathways, such as PD-1, PD-L1, and cytotoxic T lymphocyte associated antigen-4 (CTLA-4), which down-regulate T-effector cell responses, contributing to immune suppression [18]. Chemotherapeutic agents such as Paclitaxel have been shown to increase PD-L1 expression on both human and mouse ovarian cancer cells [19]. In addition, over-expression of key signaling pathway components (for example, the interlukin-6 signal transducer and activator of transcription 3 (STAT3)) that contribute to immunosuppression is observed in EOCs [20]. These proteins are also associated with chemoresistance; STAT3 expression is correlated with increased cell proliferation and drug resistance [21]. However, the relationship between these known immune modulators and Sp17 expression, a key antigenic determinant on EOC cells, has never been investigated.

In this study, we have evaluated the relationship between Sp17 expression and tumor formation in vivo using the murine EOC model induced by ID8 mouse ovarian surface epithelial cell line [22]. Sp17-expressing ID8 cells were isolated by dilution cloning, and their growth and tumor-initiating capacity was investigated in vitro and in vivo. The expressions of immune-specific molecules (such as major histocompatibility class (MHC) I molecules (MHC I), MHC II, tumor necrosis factor receptor II (TNFRII), CTLA-4, CD73, CD39) on the Sp17^+^ ID8 cells were tested, and the relationships between Sp17 expression and established molecules involved in immunosuppression and chemoresistance (such as TNFRII, STAT3 and PD-L1) was also evaluated. Our data reveal a key role for Sp17 in the progression, chemoresistance and immune suppression exhibited by EOCs.

## 2. Results

### 2.1. Sperm Protein 17 Is Expressed in a Subset of ID8 Cells In Vitro

**Figure 1:**
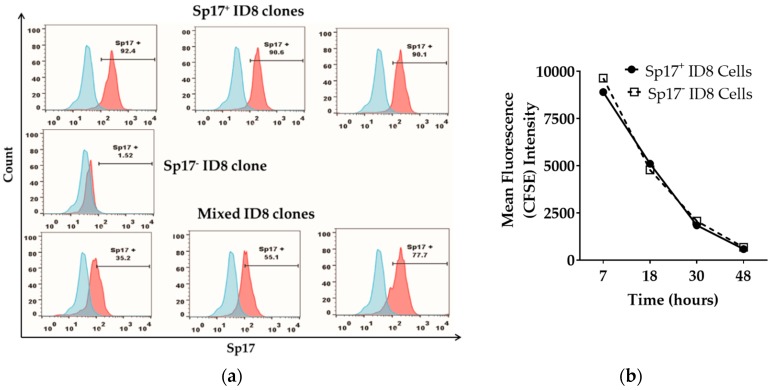
Sperm protein 17 (Sp17) is expressed in a subset of ID8 cells in vitro. (**a**) Sp17 expression in selected ID8 sub-clones: Sp17^+^ (top panel, three sub-clones obtained), Sp17^−^ (middle panel, one sub-clone obtained), and mixed clones (bottom panel, three representative sub-clones), analyzed by flow cytometry. Data is shown as histogram of Sp17 expression (red) over isotype control (blue) for each of the ID8 sub-clone presented here. The *X*-axis shows the fluorescence intensity and the *Y*-axis shows count. All cells were stained intracellularly with an anti-Sp17 antibody; (**b**) in-vitro growth of the Sp17^+^ and Sp17^−^ ID8 cells. The Sp17^+^ and Sp17^−^ cloned ID8 cells were stained by carboxyfluorescein succinimidyl ester (CFSE) and cultured for 7, 18, 30, and 48 h. CFSE fluorescence was assessed by flow cytometry at each time point.

We first evaluated the frequency of Sp17-expressing ID8 cells in vitro. Sp17 expression in ID8 cells was variable, and included cells not expressing Sp17, as well as cells clearly expressing Sp17 (Figure 1a). To further study the role of Sp17 expression in ID8 cells, we isolated Sp17-high or -low ID8 cells (or those with no Sp17) via the dilution cloning technique. Following isolation and expansion of cells in culture, we obtained 23 clonal ID8 populations for further analysis. Flow cytometry revealed significant heterogeneity amongst these clones for Sp17 expression (Figure 1b). Amongst the cell populations analyzed, three clones were clearly (>90%) positive for intracellular Sp17 expression and were designated Sp17^+^. By contrast, one clone showed no detectable staining for Sp17 (<1.6% positive cells detected); these cells were designated Sp17^−^. The remaining clonal populations were heterogeneous with respect to Sp17^+^ staining, ranging from 16% to 76% positivity, and were designated Sp17^mixed^ (Figure 1a).

To determine whether the expression of Sp17 had any influence on cell growth rate in vitro, the proliferation of Sp17^+^ and Sp17^−^ ID8 cells was assessed by a carboxyfluorescein succinimidyl ester (CFSE) assay. No difference in the proliferation rate between the two cell populations (Sp17^+^ and Sp17^−^) was observed at any time point examined, suggesting that Sp17 does not influence the in-vitro growth rate of ID8 cells (Figure 1b). Based on the highly significant differences observed in Sp17 expression between the ID8 cell sub-clones, a single population representing each of the Sp17^+^ and Sp17^−^ cells was chosen for use in subsequent in-vivo analyses.

In our study, in contrast to some previous reports [15,23], we have found that Sp17 is mainly localized in the cytoplasm of the ovarian cancer cell lines with limited positive staining of the cell surface, which has been similarly reported by Li et al. (2009) [11]. This may indicate that Sp17 can be secreted out of the cells, and surface expression is transient and unstable; alternately, surface Sp17 might bind to syndecan (or a carbohydrate complex) [24] in the culture/tissue microenvironment and escape detection by the staining antibody. However, intracellular expression of Sp17 is stably and accurately detectable by the staining antibody. Therefore, we used intracellular Sp17 staining for the determination of Sp17 expression in our study.

### 2.2. Sperm Protein 17 Expression Is Required for Tumor Formation In Vivo

**Figure 2:**
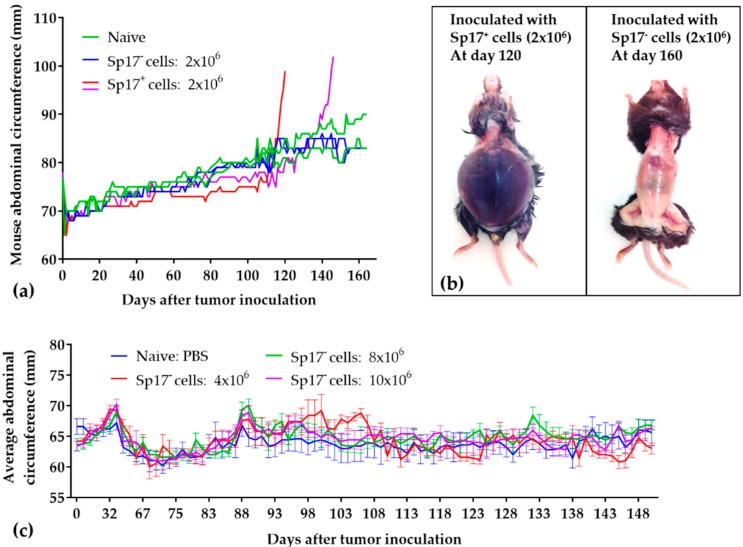
Tumorigenicity of Sp17^+^ and Sp17^−^ ID8 cells in vivo. (**a**) Direct comparison of tumorigenicity of the Sp17^+^ and Sp17^−^ ID8 cell clones. Female C57BL/6 mice (*n* = 2/group) were injected intraperitoneally with 2 × 10^6^ of Sp17^−^ ID8 cells (blue) or Sp17^+^ ID8 cells (red and purple). Negative control mice received 100 µL PBS (green). Tumor growth were determined by the changes of the mouse abdominal circumferences (mm) over a period of time. Data is shown as the daily abdominal circumference (mm) for each individual mouse measured from day 1 to the study endpoint; (**b**) Representative images of mice at cull which had received either Sp17^+^ or Sp17^−^ ID8 cells, confirming the presence/absence of tumor mass; (**c**) Additional number of Sp17^−^ ID8 cells (4 × 10^6^, 8 × 10^6^, and 10 × 10^6^ per mouse) were inoculated to female C57BL/6 mice (*n* = 5/group) intraperitoneally, but failed to induce tumors (monitored up to 150 days post inoculation). Data presented as average of mouse abdominal circumference (mm) ± SEM measured at each time point. Negative control mice received 100 μL PBS.

To determine whether Sp17 expression contributes to tumorigenicity in vivo, we inoculated mice intraperitoneally with 2 × 10^6^ Sp17^+^ or Sp17^−^ ID8 cells, and monitored tumor progression for up to 170 days. As shown in Figure 2, mice receiving Sp17^+^ ID8 cells developed tumors with 100% penetrance, exhibited significant abdominal distention, and reached an endpoint between days 90–150 post-inoculation (an initial drop in abdominal circumferences between days 80–110 may be due to the cancer associated cachexia) (Figure 2a). Extensively disseminated tumor foci were observed at autopsy throughout the abdominal cavity, with multiple macroscopic lesions on the omentum, liver, intestines and diaphragm, and up to ~10 mL ascites fluid was collected from each mouse (Figure 2b left), consistent with previous reports for the ID8 EOC model [22]. By contrast, mice receiving Sp17^−^ ID8 cells failed to form any detectible tumors within the time period examined (Figure 2a,b right).

To further test whether cell numbers might impact the formation of tumors, mice were inoculated with increased numbers of Sp17^−^ ID8 cells (4 ×, 8 ×, and 10 × 10^6^) intraperitoneally. No tumor formation by Sp17^−^ cells was observed in mice up to 150 days post-inoculation, and no difference in body circumference measurements over the monitoring period comparing to the control mice injected with phosphate-buffered saline (PBS) (Figure 2c). The data therefore indicates that Sp17 expression is associated with the formation of progressive tumor implants in vivo by ID8 cells.

### 2.3. Sperm Protein 17 Expression Correlates with Altered Immunosuppressive Potential in ID8 Cells

**Figure 3:**
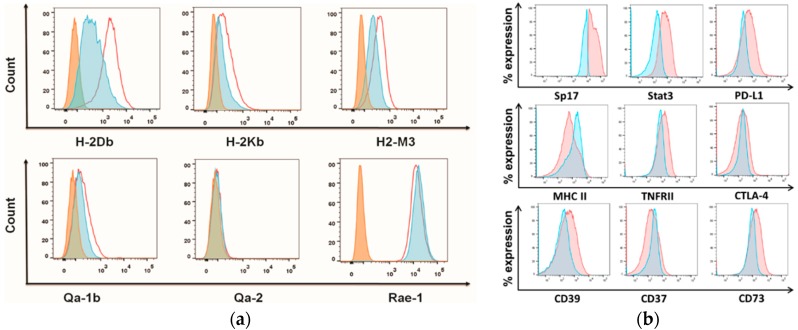
Sp17 expression modulates major histocompatibility class (MHC) expression and correlates with immunosuppressive molecules in ID8 cells. (**a**) Classical and non-classical MHC I molecule expression (H2-Db, H2-Kb, H2-M3, Qa-1b, Qa-2, and ribonucleic acid export 1 (rae-1)) in Sp17^+^ and Sp17^−^ ID8 cells. Data is shown as histograms of each marker relative expression on Sp17^+^ (red unshaded) and Sp17^−^ (blue) ID8 cells, as well as isotype control (orange); (**b**) Immunosuppressive molecules expressions (PD-L1, MHCII, CD39, CD37, CD73, cytotoxic T lymphocyte associated antigen-4 (CTLA-4), tumor necrosis factor receptor II (TNFRII), and STAT3) on gated Sp17^+^ and Sp17^−^ ID8 cells. The Sp17^+^ and Sp17^−^ ID8 cells (for Figure 3a) or un-fractionated ID8 cells (for Figure 3b) were stained with specific antibodies for each immune marker assessed here and analyzed by flow cytometry (see Materials and Methods). Data is shown as histograms for each marker-relative expression in Sp17^+^ (red) and Sp17^−^ (blue) ID8 cells, as well as isotype control (orange). The *X*-axis shows the fluorescence intensity, and *Y*-axis shows count (**a**) or % expression normalized to mode (as a percent expression relative to each population) (**b**). Results are representative of three independent experiments.

Based on the observed failure of Sp17^−^ cells to induce tumors in vivo within a given time period, we speculated that Sp17^+^ cells may down-regulate MHC I to escape immune recognition and CD8^+^ T cell-mediated target cells lysis. We therefore evaluated the co-expression of Sp17 with MHC I by flowcytometry. Unexpectedly, Sp17^+^ ID8 cells displayed increased surface expression of the classical MHC I molecules H2-Db, H2-Kb, and H2-M3, as well as the non-classical MHC I molecule Qa-1b relative to the Sp17^−^ ID8 cells (Figure 3a). Whereas, the expression of non-classical MHC I molecule Qa-2, as well as ribonucleic acid export 1 (RAE-1, one of the natural killer group-2 member D (NKG2D) ligands) were relatively unchanged (Figure 3a). Together, this data suggests that the overall MHC I expression in ID8 cells may not have a direct impact on cell tumorigenicity. In contrast, MHC II was down-regulated in Sp17^+^ cells compared to Sp17^−^ cells among the unsorted ID8 cells (Figure 3b). We further evaluated the co-expression of Sp17 with known immune markers associated with immunosuppression, including STAT3, PD-L1, CD39, CD37, CD73, CTLA-4, and TNFRII. Significantly, both PD-L1 and STAT3 were co-expressed in Sp17^+^ ID8 cells (Figure 3b). Compared to Sp17^−^ ID8 cells, cells expressing Sp17 also displayed increased levels of TNFRII, as well as CD39 and CD73 molecules, which are related to immunosuppression through the formation of adenosine diphosphate and adenosine [25,26]. There were no apparent correlations between Sp17 expression and CD37 or CTLA-4 (Figure 3b).

**Figure 4:**
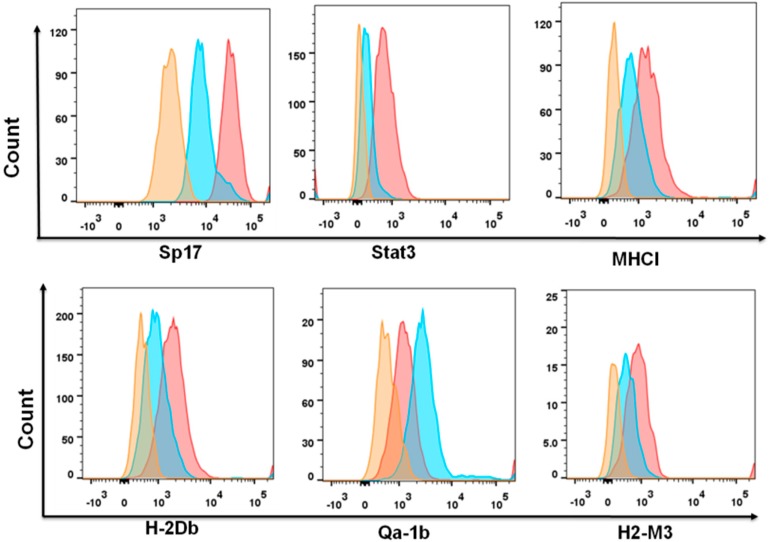
Sp17 and immune marker expression differs between PD-L1^+^MHCII^−^ and PD-L1^−^MHCII^+^ populations of ID8 cells. Original (non-clonal selected) ID8 cells were collected and stained for the expression of PD-L1, MHCII, Sp17, STAT3, H2-Db, Qa-1b, and H2-M3. Marker expression between PD-L1^+^MHCII^−^ (red) and PD-L1^−^MHCII^+^ (blue) populations was analyzed (isotype control, orange). Data is shown as histograms of each marker expression between these two populations.

To confirm the relationships between Sp17 and immune-related molecules, we further isolated ID8 cells according to PD-L1 and MHC II surface expression by flow cytometry, and compared their expression of Sp17 and STAT3, as well as several related molecules, such as MHC I, H2-Db, H2-M3, and Qa-1b (Figure 4). As expected, PD-L1^+^MHCII^−^ cells were also Sp17^+^STAT3^+^, with upregulation of MHC I molecules H-2Db, H2-M3, and Qa-1b.

### 2.4. PD-L1^high^MHCII^−^ (Sp17^high^) Cells Display Increased Resistance to Paclitaxel In Vitro

**Figure 5:**
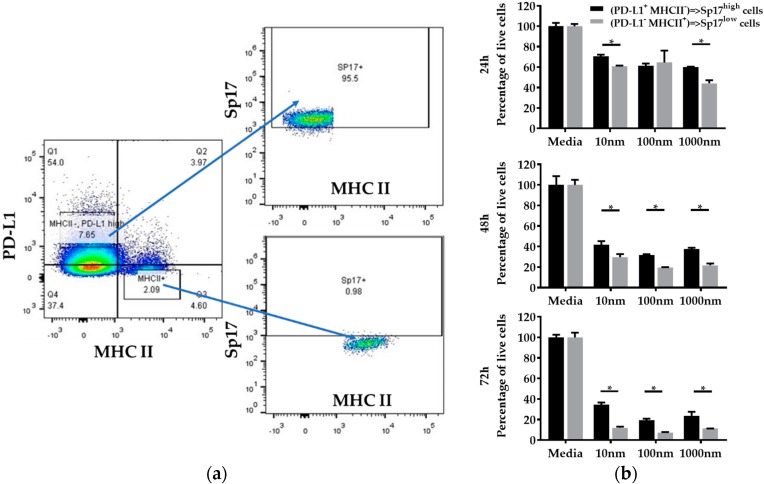
PD-L1^high^MHCII^−^ (Sp17^high^) population of ID8 cells are more Paclitaxel-resistant than PD-L1^−^MHCII^+^ (Sp17^low^) populations. (**a**) ID8 cells were FACS-sorted into two populations: PD-L1^high^MHCII^−^ and PD-L1^−^MHCII^+^, based on cell surface staining with anti-PD-L1 and anti-MHC II antibodies, which represented the Sp17^high^ and Sp17^low^ cells. (**b**) MTT viability assay was used to assess the cytotoxicity of the two populations at multiple time points (24 h, 48 h, and 72 h) and doses (10–1000 nM). Data show the mean percentage of live cells from four samples with standard deviation for each condition. * *p* < 0.05 (unpaired, two-tailed Student’s *t*-test).

Both STAT3 and PD-L1 expression are associated with chemoresistance in ovarian cancers [19,21,27]. Based on the observed co-expression of Sp17 with STAT3 and PD-L1, we isolated ID8 cells by fluorescence-activated cell sorting (FACS) for PD-L1^high^MHCII^−^ and PD-L1^−^MHCII^+^ phenotypes, representing the Sp17^high^ population (>95% Sp17^+^) and Sp17^low^ population (<1% Sp17^+^), respectively (Figure 5a), and examined their responses to Paclitaxel in vitro. After sorting, both PD-L1^high^MHCII^−^ (Sp17^high^) and PD-L1^−^MHCII^+^ (Sp17^low^) cells were grown to 80% confluence, and then incubated for up to 72 h in the presence of Paclitaxel (doses from 10 nM to 1000 nM) prior to the determination of cell viability by MTT (3-(4,5-dimethylthiazol-2-yl)-2,5-diphenyltetrazolium bromide) assay. After 72 h, both cell populations displayed a significant reduction in the number of viable cells; however, percentage survival was about three-fold higher for PD-L1^high^MHCII^−^ cells (the Sp17^high^ population) compared to the PD-L1^−^MHCII^+^ cells (the Sp17^low^ population) (Figure 5b), suggesting that the Sp17^high^ cell population displayed increased resistance to chemotherapy. Similar results were obtained at each dose of Paclitaxel tested (Figure 5b).

## 3. Discussion

In this study, we identified a key association between expression of the CTA Sp17 and tumor progression. Several tumor types (including ovarian) are known to express elevated levels of Sp17 [28,29,30], and multiple studies have suggested Sp17 as an antigenic target for the development of therapeutic vaccines [17,31,32]. However, no studies have previously compared the tumorigenicity or phenotypic differences between EOC cell clones or lines with different levels of Sp17 expression. The results shown here indicate Sp17 expression is required for the formation of ID8 tumors in vivo. Concurrently, Sp17^+^ cells also displayed enhanced chemoresistance to Paclitaxel, a standard first-line chemotherapy for EOC [33]; and expressed increased levels of multiple markers, including STAT3 and PD-L1, respectively linked to chemoresistance and immune suppression [19,27]. Sp17 is highly expressed in EOC [12,15,30,34]. Chiriva-Internati et al. (2002) report that Sp17 is expressed in 70% of OC patients [34]. Straughn et al. (2004) show that Sp17 transcripts were detected in 83% of primary ovarian tumors, while Sp17 mRNA and protein were also detected in 68% and 42% of OC patients, respectively [15]. Nakazato et al. (2007) show that Sp17 is highly expressed (7/7, 100%) in clear cell carcinomas of the ovary [12]. Li et al. (2010) further show that Sp17 protein is aberrantly expressed in 43% of the patients with primary epithelial OC, 100% in the metastatic cancer cells of ascites, and 100% in clear cell OC [30]. Together, these data show an association of Sp17 with ovarian malignancy. Expression of Sp17 has reportedly been detected in normal ciliated epithelia cells [35], particularly in the motile cilia of the fallopian tube, but not in the non-ciliated cells lining the oviduct epithelium, suggesting that Sp17 might be involved in the motion movement function of the cells and transport fluid or materials over epithelia [35]. However, the potential involvement of Sp17 in cancer oncogenesis is yet to be elucidated. Our data suggests that Sp17 expression may play a key role in tumor progression and response to therapy in vivo.

The ability of ID8 cells to establish progressive tumors in C57BL/6 mice was dependent on Sp17 expression. This phenomenon was not related to cell proliferation, as both Sp17^+^ and Sp17^−^ cell populations displayed identical proliferation rates in vitro; moreover, the inability of Sp17^−^ cells to form tumors was not alleviated by increased cell load during inoculation. We also noted that expression of some classical MHC class I molecules (H2-Db, H2-Kb, and H2-M3) was increased in the tumor-forming Sp17^+^ cell population, but expression of non-classical MHC I molecules (e.g., Qa-2) remained unchanged, as did the expression of rae-1, a ligand for the NK cells stimulatory receptor NKG2D. Classical MHC I molecules present antigenic peptide ligands on infected cells to CD8^+^ T cells, whereas a key function for non-classical MHC I molecules is to mediate inhibitory or activating stimuli in natural killer (NK) cells. Therefore, while increased classical MHC I expression can promote recognition by cytotoxic T lymphocytes, it could also decrease NK cell recognition [33,36,37,38], and has previously been associated with poor patient prognosis [39]. Regardless of potential effects on NK cells, the observation of MHC I expression on Sp17^+^ ID8 cells suggests they do not have impaired MHC I-mediated antigen presentation to CD8 T-cells. Sp17 expression has also been linked to enhanced migratory potential in ovarian cancer cell lines [11]. Taken together, our data suggest that Sp17 expression is a determinant of tumorigenicity in the ID8 EOC model. It is important to note that we only considered tumor-forming potential in immune-competent mice; whether the Sp17^−^ population is also able to form progressive tumors in an immune-suppressed model is currently unknown.

The immune-reactive nature of EOCs has been well documented, particularly the established involvement of tumor-infiltrating T-cells in tumor progression and their correlation with overall survival [40]. However, several immune evasion strategies are employed in EOCs, ultimately leading to escape from immune surveillance [41,42,43]. Whilst the Sp17^+^ cell subset retained MHC I expression, it down-regulated MHC II and was enriched in both STAT3 and PD-L1 expression. A well-established driver of tumorigenesis in multiple solid tumors, STAT3 induces and maintains a pro-inflammatory environment during tumor initiation and progression, and is aberrantly activated in EOCs [44]. Over-expression of STAT3 is associated with the invasive and metastatic potential of EOCs (reviewed [44]). Similarly, PD-L1 expression is associated with tumor grade and is negatively correlated with survival in patients with advanced EOC [45,46]. Moreover, PD-L1 expression is also associated with the peritoneal dissemination of EOC through host immune suppression [47]. The observation that Sp17^+^ cells are required to initiate tumor formation in the ID8 model suggests that STAT3 and PD-L1 expression may be key to this process, and that Sp17 may act as a surrogate marker for the presence of these immunosuppressive, metastatic cells in tumors. PD-L1 transcription is regulated by STAT3 [48], suggesting STAT3 may be the primary driver for PDL-1 upregulation in our system. However, thus far there is no reported evidence linking Sp17 to the STAT3 signaling pathway, which may open up a new and exciting area of research.

Previous studies have demonstrated the Paclitaxel-mediated induction of PD-L1 expression in ovarian and other solid tumor types [19,49], as well as a potential correlation between Sp17 expression and chemoresistance in clear cell ovarian carcinoma [12]. The induction of PD-L1 by paclitaxel occurs in a nuclear factor-κB (NF-κB)-dependent manner [19], suggesting that Sp17^+^ cells may exhibit increased NF-κB signaling. Interestingly, previous work also established that PD-L1 induction is transient; 2–5 days following Paclitaxel administration, PD-L1 levels return to baseline, both in cell culture and in patients receiving chemotherapy [19]. We did not examine time-dependent PD-L1 expression in this study; however, we noted that Sp17 expression was dynamic in cell cultures. Indeed, the Sp17^−^ population acquired low levels of Sp17^+^ cells over time; conversely, the Sp17^+^ population showed a reduction in Sp17 expression following prolonged culture (data not shown). These results suggest that the Sp17^+^PDL1^+^STAT3^+^ population is transient, and can increase to promote tumorigenicity in certain circumstances—for example, under selective pressure imposed by chemotherapy.

Activated STAT3 is over-expressed in a majority of Paclitaxel-resistant ovarian cancer cells and tumor tissues, and thus represents an important target for anti-tumor therapies [45,50]. STAT3 activation by Paclitaxel, via the induction of reactive oxygen species, has been demonstrated in several tumor types [51]; however, this phenomenon is cell-type-specific, and conflicting effects on STAT3 expression in response to Paclitaxel have been reported in ovarian cancers [52,53,54]. We did not determine STAT3 activation status; thus, while expression is associated with the Sp17^+^ phenotype, it is not clear whether STAT3 activation accompanies its enrichment following chemotherapy. However, STAT3 activation does lead to the down-regulation of MHC class II molecules [55,56,57], consistent with our observation that Sp17^+^STAT3^+^cells displayed an MHCII^−^ phenotype.

High-grade EOCs demonstrate significant increase in PD-L1 gene expression, which is associated with poor prognoses [58,59,60]. Our finding suggests that the combination of Paclitaxel and anti-PD-L1 could synergize to specifically target the Sp17^+^ tumor-initiating cell population. Accordingly, Paclitaxel chemotherapy in PD-L1-ovarian tumors leads to reduced tumor burden in vivo and increased tumor cell lysis in vitro [19]. Nevertheless, the majority of pre-clinical studies have evaluated combinations of cisplatin and anti-PD1 therapy [61,62,63], and anti-PD-L1 therapies have not been similarly evaluated. Clinical trials have also focused on administration of anti-PD-1, which has shown only modest effects in ovarian cancer patients; a recent trial assessing response to anti-PD-L1 in ovarian cancer obtained a 9.7% response rate and 44% stable disease rate amongst patients with platinum-resistant disease (clinical trial NCT01772004) [64]. Several other trials evaluating anti-PD-L1 therapy are currently in progress (reviewed in [2]). It will be important to determine whether Paclitaxel therapy results in the sustained enrichment of tumor-specific PD-L1-expression in vivo, and to evaluate its potential impact on the efficacy of anti-PD-L1 therapy.

In conclusion, intracellular Sp17 expression identified a sub-population of ID8 ovarian cancer cells with tumor-forming potential, which display increased expression of PD-L1 and STAT3. The co-expression of Sp17 with PD-L1 and STAT3 suggests that Sp17 might prove a useful surrogate marker to indicate susceptibility to immune checkpoints or STAT inhibitors. These therapies, in combination with Paclitaxel, may therefore prove useful in specifically targeting the Sp17^+^ tumor-initiating cells in ovarian cancers, potentially including vaccines, given the demonstrated capacity of this tumor-associated antigen as the target of protective and therapeutic immune responses in pre-clinical animal models [17,32].

## 4. Materials and Methods

### 4.1. Isolation of Sp17^+^ ID8 Clones

ID8 cells (a gift from Dr. Katherine F. Roby, Department of Anatomy and Cell Biology, University of Kansas Medical Centre, Kansas City, KS, USA) were cultured in complete media (Roswell Park Memorial Institute medium (RPMI 1640)) supplemented with 10% fetal bovine serum (FBS), 20 mM HEPES (4-(2-hydroxyethyl)-1-piperazineethanesulfonic acid), 4 mM L-glutamine, 0.1 mM 2-mercaptoethonal, 100 units/mL penicillin, and 100 μg/mL streptomycin at 37 °C 5% CO_2_. The Sp17 positive and negative cell populations were isolated via dilution cloning. Briefly, ID8 cells were seeded at theoretically 0.5 cells per well into a 96 well U-bottom plate, and cultured until cell growth was observed. Outgrown cells were trypsinized, washed, and transferred to six-well cell culture plates and further T25 flasks for expansion. Cell clones were stained intracellularly with anti-Sp17 antibodies, and Sp17 expression was determined by flow cytometry.

### 4.2. ID8 Murine Ovarian Cancer Model

The murine C57BL/6 ID8 EOC model is a syngeneic mouse model developed from mouse ovarian surface epithelial cells (MOSEC) [22], which closely mimics the most lethal type of spontaneous human EOC. C57BL/6 female mice, 6–8 weeks of age, were purchased from Monash Animal Services (Clayton, VIC, Australia). All experiments were approved by the Alfred Medical Research and Education Precinct (AMREP) animal ethics committee, Melbourne, Australia (approval #E/1051/2011/M). Treatment and care of the animals were in accordance with Institutional Guidelines and the Animal Welfare Assurance Act.

Tumors were established in C57BL/6 mice by intraperitoneal (IP) injection of 2–10 × 10^6^ ID8 cells per animal. Mice were monitored for weight, abdominal circumference, general wellbeing, and overall survival. The endpoint was determined by the mouse body condition scores for general wellbeing and body abdominal circumference (maximum 10 cm), according to approved ethics.

### 4.3. Flow Cytometry

Flow cytometry was carried out as described [21]. Cells were stained with fluorochrome-conjugated mAb against PD-L1 (PE), STAT3 (APC), MHC I (biotin), H2-Db (biotin), Qa-1b (biotin), H2-Kb (PE), CTLA-4 (PE), mouse IgG1 (FITC), Streptavidin (Percp) (BD Pharmingen, CA, USA), MHC II (APC-Cy7) (eBioscience, Vienna, Austria), CD39 (PE-Cy7) (eBioscience, Vienna, Austria), CD73 (biotin) (eBioscience, Vienna, Austria), TNFRII (biotin) (eBioscience, Vienna, Austria), and Sp17 antibodies (unconjugated) (SC-365325, Santa Cruz, CA, USA). Surface staining was performed on 0.5 × 10^6^ cells/well in “V-bottom” plates, with 20 μL of antibody-staining cocktail (at predetermined concentrations, diluted in PBS with 2% FBS). Plates were incubated on ice for 30 min. After incubation, cells were washed twice by additional 100 μL/well of washing buffer (PBS/2% FBS) and centrifuged at 1500 rpm for 5 min to remove the supernatant. The addition of secondary antibodies (such as anti-mouse IgG1-FITC or anti-rabbit IgG-FTIC) were also added to the unlabeled antibodies as appropriate, and the above procedures were repeated. Finally, the LIVE/DEAD aqua fixable dead cell stain (Invitrogen, Carlsbad, CA, USA) was added following the manufacturer’s instructions. For intracellular staining, fixation and permeabilization was performed using a fixation/permeabilization kit (eBioscience, San Diego, CA, USA) as described by the manufacturer. Intracellular antibodies (anti-STAT3 and an anti-Sp17) were diluted in a permeabilization wash buffer. Following staining, cells were washed with the permeabilization wash buffer and fixed in 1% paraformaldehyde (PFA).

Labelled antibody samples were analyzed using a four laser LSRII flow cytometer (Becton Dickinson, Franklin Lakes, NJ, USA) with FACS Diva software (Becton Dickinson, Franklin Lakes, NJ, USA), or using a FACS ARIA flow cytometer (Becton Dickinson, Franklin Lakes, NJ, USA) when cell sorting was required. Fluorescence minus one (FMO) and isotype antibodies were used for staining controls to ensure gating accurately. All flow cytometry data were analyzed using FlowJo7.6.5 software (TreeStar, Ashland, OR, USA).

### 4.4. Cytotoxicity Assay

Cytotoxicity in the presence of Paclitaxel was tested by an MTT (3-(4,5-dimethylthiazol-2-yl)-2,5-diphenyltetrazolium bromide) assay. ID8 cells (1 × 10^5^ cells/100 μL/well) were cultured in a 96-well flat-bottomed plate, and incubated overnight at 37 °C. Cells were then treated with Paclitaxel (10, 100 and 1000 nM) for 24, 48 or 72 h at 37 °C 5% CO_2_. On completion of the incubation period, 5 μL of 5 mg/mL MTT solution was added to each well and incubated for 4 h at 37 °C. Cells were then harvested and washed once in PBS by centrifugation in the culture plate. Finally, 150 μL/well of DMSO were added to the cells and incubated for 10 min (with shaking), and the absorbance of each well was measured at 590 nm wavelength on a Multiskan GO microplate reader (Thermo Scientific, Waltham, MA, USA).

### 4.5. Carboxyfluorescein Succinimidyl Ester Proliferation Assay

ID8 cell proliferation was determined using carboxyfluorescein succinimidyl ester (CFSE) [65]. Briefly, 4 × 10^6^ cells were incubated with 0.8 µM CSFE for 10 min at 37 °C. Staining was quenched by the addition of ice-cold RPMI 1640 supplemented with 10% FBS. Cells were then washed in PBS once and recovered by centrifugation at 1500 rpm, 5 min at 4 °C. Labelled cells (1 × 10^6^ cells per time point) were cultured in complete media in T25 flasks for periods of 7, 18, 30, and 48 h prior to fixation in 1% PFA, and the intracellular fluorescence was detected and analyzed by flow cytometry.

### 4.6. Statistical Analyses

All statistical analyses were performed using Graph Pad Prism v6.04 software (Graph Pad Software, Inc., La Jolla, CA, USA) and Microsoft Excel (Microsoft Corporation, Redmond, Washington, DC, USA). Comparisons were performed using one-way or two-way ANOVA or an unpaired two-tail Student’s *t*-test. Differences were considered statistically significant when *p* < 0.05. Values are expressed as mean ± standard deviation (SD).

## 5. Conclusions

In conclusion, Sp17 plays an important role in EOC formation and disease progression. Our data shows that Sp17 expression is required for the EOC tumor formation in vivo, and correlates with altered immunosuppression potential in ID8 cells. Furthermore, Sp17 expression cells are also associated with the expressions of PD-L1 and STAT3 and display increased resistance to paclitaxel in vitro. Together, our data suggest that Sp17 is an ideal target for vaccine development and therapeutic treatment of ovarian cancer. Therapeutically targeting Sp17 expression tumor-initiating cells in ovarian cancer, in combination with Paclitaxel or antibodies to checkpoints inhibitors, could be a powerful immunotherapeutic strategy for the treatment of ovarian cancer.

## 6. Patents

Title: T and B cell epitopes in surface sperm protein 17 as cancer vaccines, WO/2017/013231; PCT/EP2016/067468.

## Abbreviations

HEPES	2-[4-(2-Hydroxyethyl)-1-piperazinyl] ethanesulfonic acid
MTT	3-(4,5-Dimethylthiazol-2-yl)-2,5-Diphenyltetrazolium Bromide
CTAs	Cancer testes antigens
CD	Clusters of Differentiation
CTLA-4	Cytotoxic T lymphocyte associated antigen-4
EOC	Epithelial ovarian cancer
FACS	Fluorescent-activated cell sorting
Her2	Human epidermal growth factor receptor 2
MHC I	Major histocompatibility class I molecules
MUC16	Mucin 16
NHMRC	National health and medical research council
NK	Natural killer
NY-ESO-1	New York esophageal squamous cell carcinoma 1
OC	Ovarian cancer
PBS	Phosphate buffered saline
PD-L1	Programed cell death ligand-1
PD-1	Programed cell death protein-1
RPMI	Roswell Park Memorial Institute
STAT3	Signal transducer and activator of transcription 3
Sp17	Sperm protein 17
TNFRII	Tumor necrosis factor receptor II
TP53	Tumor protein p53
